# The association between exposure to air pollutants and latent tuberculosis infection prevalence in the elderly population: a population-based cross-sectional study from China

**DOI:** 10.7189/jogh.16.04116

**Published:** 2026-04-03

**Authors:** Yaqi Zhao, Haitao Li, Yijun He, Xiaoyan Guo, Jin Jin, Taifeng Li, Shuping Wang, Xuesong Cao, Yunfeng Deng, Boxuan Feng, Zihan Li, Qian Wang, Shan Cao, Zhonghui Zhao, Xiaoguo Zhang, Aihua Zhu, Jianguo Liang, Hong Geng, Yong Sang, Jing Li, Li Wang, Xiankun Fan, Henan Xin, Jiang Du, Xuefang Cao, Zhongfa Zhang, Lei Gao

**Affiliations:** 1 National Health Commission of the People’s Republic of China Key Laboratory of Systems Biology of Pathogens, National Institute of Pathogen Biology, and Centre for Tuberculosis Research, Chinese Academy of Medical Sciences and Peking Union Medical College, Beijing, China; 2 Key Laboratory of Pathogen Infection Prevention and Control, Ministry of Education, National Institute of Pathogen Biology, Chinese Academy of Medical Sciences and Peking Union Medical College, Beijing, China; 3 Tuberculosis Centre, Shandong Public Health Clinical Centre, Jinan, China; 4 Tuberculosis Prevention and Control Department, Shandong Provincial Centre for Disease Control and Prevention, Jinan, China; 5 Tuberculosis Department, Liaocheng Infectious Disease Hospital, Liaocheng, China; 6 Tuberculosis Prevention and Control Department, Weihai Centre for Disease Control and Prevention, Weihai, China; 7 Tuberculosis Prevention and Control Department, Weihai Huancui District Tuberculosis Prevention and Treatment Institute, Weihai, China; 8 Tuberculosis Prevention and Control Department, Disease Control and Prevention Centre of Dongchangfu District, Liaocheng, China

## Abstract

**Background:**

Globally, research on the direct correlation between air pollutants and latent tuberculosis (TB) infection (LTBI) is still relatively scarce. We aimed to conduct a cross-sectional study of LTBI across regions with varying air quality to assess whether air pollution affects the burden of TB infection.

**Methods:**

We selected the cities of Liaocheng and Weihai in Shandong Province, China, as the research sites from 13–30 April 2025. We used the γ-interferon release assay to detect LTBI. We assessed the concentrations of particulate matter (PM)_2.5_, PM_10_, carbon monoxide (CO), nitrogen dioxide (NO_2_), sulphur dioxide, and ozone across various time periods by integrating satellite remote sensing data with ground-based monitoring data. We used logistic regression and weighted pollution models to assess the relationship between air pollutants and the prevalence of LTBI.

**Results:**

A total of 2504 participants were included in the study, and 271 latent infected individuals were identified. The LTBI prevalence in Liaocheng city and Weihai city were 11.8% and 9.7%, respectively. During the three-year exposure window, for every 10 μg/m^3^ increase in PM_2.5_, the odds of LTBI were 35.0% higher in Liaocheng city (odds ratio (OR) = 1.35; 95% confidence interval (CI) = 1.04–1.78) and 6.0% higher in Weihai city (OR = 1.06; 95% CI = 1.03–1.09). For every 10μg/m^3^ increase in NO_2_ concentration, the odds of LTBI in Liaocheng city was 35% higher than in Weihai city (OR = 1.50 *vs.* OR = 1.15). The results of the weighted pollution model indicated that the comprehensive exposure index was positively correlated with the LTBI prevalence. The subgroup analysis results indicated that the association between exposure to PM_2.5_, PM_10_, CO, and NO_2_ and the prevalence of LTBI was more pronounced in populations with a household per capita income < 5000 RMB, daily ventilation time < 0.5 hours, and the use of non-renewable energy for cooking (*P* < 0.05).

**Conclusions:**

The research indicates that prolonged exposure to air pollutants substantially elevates the odds of LTBI in Chinese cities, exhibiting regional disparities. This underscores the significance of managing air pollution to prevent and control TB.

Tuberculosis (TB) is a serious infectious disease that poses a significant threat to human health and is transmitted through the air [1,2]. It is estimated that nearly one quarter of the global population is in a latent tuberculosis infection (LTBI) state [3]. LTBI of *Mycobacterium tuberculosis* (MTB) represents a substantial reservoir of potential ‘patients’ [4]. The State of Global Air 2020 Report indicates that in 2019, air pollution became the fourth leading risk factor for mortality worldwide [5,6]. Currently, most studies on air pollutants and TB have focused on the risk of active pulmonary TB [7]. Nonetheless, some studies have provided indirect evidence. A multi-centre prospective cohort study [8] conducted in rural areas of China (n = 20 869) found that long-term exposure to pollutants such as particulate matter (PM)_2.5_, PM_10_, sulphur dioxide (SO_2_), and nitrogen dioxide (NO_2_) was associated with the development of active TB in patients with LTBI. A nested case-control study conducted in the USA, along with two recent systematic review meta-analyses, has reported that exposure to single or mixed gaseous pollutants (such as SO_2_, NO_2_, carbon monoxide (CO), and ozone (O_3_)) in air pollutants is significantly associated with an increased risk of TB [7,9,10]. This may be related to environmental air pollution interfering with the lung's immune barrier, reducing its defence and immune functions and thereby promoting the occurrence and progression of lung diseases [11].

However, to date, there has been a lack of direct research on the association between inhalable particulate matter (PM_2.5_, PM_10_) and gaseous pollutants (SO_2_, NO_2_, CO, O_3_, *etc.*) and LTBI. At the same time, China is a country with a high burden of TB, and the characteristics of the LTBI population, as well as the levels of air pollution, differ from those in countries with a low TB burden [12]. Therefore, it is crucial to explore the association between long-term exposure to air pollutants and the prevalence of LTBI in cities with varying levels of air pollution in China. This exploration is of great significance for developing appropriate TB prevention and control strategies for our country.

## METHODS

### Study design and participants

This study was based on baseline cross-sectional data nested within an ongoing prospective cohort study. This cohort was established in April 2025 to investigate the long-term impact of environmental exposures on the progression of LTBI to active TB. Based on differences in exposure to air pollutants, we selected two representative cities in Shandong Province, China, Liaocheng and Weihai, for permanent residents aged ≥ 65 years from 13–30 April 2025.

Liaocheng is a typical inland industrial city dominated by heavy industry and coal-fired heating, where ambient air pollutant concentrations have long remained relatively high. In contrast, Weihai is a coastal city with a temperate maritime climate and a tertiary-industry-oriented economy (*e.g.* tourism and fisheries). Benefiting from favourable atmospheric diffusion conditions and limited industrial emissions, Weihai has maintained consistently low ambient air pollutant levels. This distinct pollution disparity between the two cities establishes a robust exposure gradient, which is essential for the comparative analysis of the association between air pollution and population health outcomes. A questionnaire survey was utilised to gather demographic data from the research participants, including age, gender, height, weight, family income, educational level, smoking and drinking habits, among other factors. Additionally, information on indoor air pollution, including ventilation practices and fuel types used for heating and cooking, was collected.

Inclusion criteria for research participants were: individuals aged ≥ 65 years; permanent residents with local household registration (living in the local area for more than six months annually); and willingness to sign an informed consent form and ability to cooperate with the study. Exclusion criteria were: individuals with active TB; pregnant or planning to become pregnant women; and those with mental disorders, deaf-mute disabilities, or mobility impairments that prevent cooperation with the test (their daily activities, particularly outdoor mobility, were severely restricted, resulting in substantial discrepancies in ambient air pollution exposure levels between this group and the general population. Meanwhile, such individuals face great difficulties in cooperating with on-site γ-interferon release assay (IGRA) testing and questionnaire surveys, which are likely to lead to data missingness and measurement bias) or whose judgment by the researchers indicates they are unsuitable for participation in this study (participants with other serious illnesses face a heightened risk of being lost to follow-up and thus failing to complete the two-year follow-up period).

Ethics committees of the Institute of Pathogen Biology, Chinese Academy of Medical Sciences, approved the study protocol (approval number IPB-2025-01).

### The identification of LTBI

Professional medical staff who have undergone unified training collect 4 mL of venous blood from subjects using a collection tube containing lithium heparin as an anticoagulant. We conducted the IGRA test using an enzyme-linked immunosorbent assay to measure γ-interferon concentration. The QuantiFERON Gold In-Tube (QFT-GIT) kit (Qiagen, Hilden, Germany), which has been uniformly purchased, was employed. In the current study, we defined TB infection as a positive result on the baseline QFT-GIT test, with a cut-off value of 0.35 IU/ml or higher [13].

### Assessment of exposure to air pollution

The air pollutants data used in this study mainly come from the widely applied China High Resolution High-Quality Near-Surface Air Pollutants Data set (China High Air Pollutants) [14–17]. The data set was developed using artificial intelligence to address spatial gaps in the moderate resolution imaging spectroradiometer multi-angle implementation of atmospheric correction aerosol optical depth satellite products. It integrates ground-based observations, atmospheric reanalysis, and emission inventories to generate comprehensive ground-level data for PM_2.5_, PM_10_, CO, NO_2_, SO_2_, and O_3_ from 2000 to the present. For PM_2.5_, PM_10_, and O_3_, the spatial resolution is 1km, and the 10-fold cross-validation determination coefficient (CV-R^2^) is 0.92, 0.90, and 0.89, respectively. The verification results are of high quality. For other gaseous pollutants, prior to 2019 the spatial resolution was 10 km; since 2019, it has been 1 km. The CV-R^2^ values for CO, NO_2_, and SO_2_ pollutants are 0.80, 0.93, and 0.84, respectively. By establishing a correlation between the latitude and longitude coordinates of residential locations and the nearest pollutant data grid cells, we were able to match the pollutant data grid cells to the home addresses of each participant. We estimated pollutant concentrations for each research participant during four distinct exposure periods, including the daily average values of pollutants for the previous one-year, three-year, five-year, and 10-year periods preceding the measurement date, to ensure data reliability. The one to three-year short-term window corresponds to the time scale of respiratory mucosal barrier damage and acute immunosuppression induced by air pollution, and thus reflects the impact of recent exposure on the primary infection of MTB. The five-year medium-term window aligns with the process of chronic inflammation-mediated immune imbalance, which is directly linked to an elevated risk of LTBI reactivation. The 10-year long-term window matches the cumulative effects of irreversible immune impairment caused by sustained exposure to air pollution and corresponds to the ‘long-term latency-slow progression’ characteristic of TB infection [8].

### Statistical analysis

Two professionals independently entered the data using EpiData, version 3.1 (The EpiData Association, Odense, Denmark), by data entry clerks. Any discrepancies between the two data sets were verified by cross-comparing them with the original data to ensure their accuracy. Statistical analysis was conducted using *R*, version 4.4.3 (R Core Team, Vienna, Austria), and GraphPad Prism, version 10.1.2 (GraphPad Software, San Diego, California, USA), for analysis and plotting. We calculated the body mass index (BMI) by dividing an individual’s weight in kilograms by the square of their height in meters. BMI < 18.5 kg/m^2^ was considered low weight; 18.5 ≤ BMI < 24 kg/m^2^ was considered normal weight; 24 ≤ BMI < 28 kg/m^2^ was considered overweight; and BMI ≥ 28 kg/m^2^ was considered obese [18]. Current drinking status was defined based on the questionnaire survey of participants’ alcohol consumption over the past year. Drinkers were considered participants who consumed alcohol at least once per week in the past year. Categorical variables were described as frequencies and percentages, whereas non-normally distributed continuous variables were summarised using the median (MD) and interquartile range (IQR). The normality of continuous variables was assessed using the Shapiro-Wilk test. For normally distributed continuous variables, comparisons between two groups were performed using the independent-samples *t* test. For non-normally distributed continuous variables, we employed the Mann-Whitney U test. All statistical tests were two-tailed, with the significance level set at α = 0.05. We used the χ^2^ test to assess whether the distributions of various characteristics differed across regional populations. We employed the variance inflation factor (VIF) to assess multicollinearity among the six air pollutants included in the study. A VIF > 10 was defined as the threshold for identifying severe multicollinearity. The test results indicated that all six pollutants had VIF > 10, confirming substantial multicollinearity. To avoid bias in regression coefficient estimates induced by multicollinearity, separate single-pollutant logistic regression models were constructed to evaluate the independent associations of each pollutant with LTBI. Prior covariates were used for variable selection (including age, gender, educational level, household per capita income, BMI, smoking, drinking, number of scars, self-reported history of immune diseases, self-reported history of close contact with patients, daily indoor time, daily ventilation time, presence of green plants indoors, cooking fuel, and heating method). We used multivariate logistic regression to estimate adjusted odds ratios (ORs) and 95% confidence intervals (CIs) for LTBI across exposure windows of one, three, five, and 10 years for pollutants one, three, five, and 10. We constructed a weighted pollution model to derive a weighted pollution index (WPI) and assess its predictive relationship with LTBI prevalence. We utilised WPI as a comprehensive exposure indicator, incorporating the weighted exposure levels of PM_2.5_, PM_10_, CO, NO_2_, SO_2_, and O_3_, to verify its exposure-response relationship with LTBI. Simultaneously, a quantile analysis (Q1, Q2, Q3, Q4, Q5) was employed to reveal the nonlinear association. Additionally, we conducted subgroup analyses based on prior covariates to explore correlations between air pollutants and their effects across populations. In the sensitivity analysis, we assessed the cumulative average levels of air pollutants throughout participants’ residency and categorised them into four groups (Q1, Q2, Q3, Q4), from lowest to highest. A trend test was employed to analyse the dose-response relationship between the exposure gradient and the odds of LTBI, and to determine the robustness of the research findings.

## RESULTS

### The basic characteristics of the study participants in the analysis

A total of 2504 participants were included in the final analysis across the two research sites. Among the included participants, 55.7% (n = 1394 were female and 60.6% (n = 1518) were aged ≥ 70 years. Further, 32.3% (n = 808) of the participants were current or former smokers. Additionally, 19.4% (n = 487) of the participants were drinkers. More than half of them (n = 1451) had a daily ventilation time of 0.5–4 hours. The proportion of households with a per capita income of ≥ 5000 RMB in Weihai (86.3%) was significantly higher than that in Liaocheng (29.1%) (*P* < 0.001) (**Table 1**).

**Table 1:** Baseline characteristics of the survey subjects, n (%)

Characteristics	Total (n = 2504)	Liaocheng (n = 1342)	Weihai (n = 1162)	*P*-value
Gender				0.498
*Female*	1394 (55.7)	756 (56.3)	638 (54.9)	
*Male*	1110 (44.3)	586 (43.7)	524 (45.1)	
Age in years				<0.001
*< 70*	986 (39.4)	492 (36.7)	494 (42.5)	
*≥ 70*	1518 (60.6)	850 (63.3)	668 (57.5)	
Education				<0.001
*No schooling*	809 (32.3)	782 (58.3)	27 (2.3)	
*Primary school or higher*	1695 (67.7)	560 (41.7)	1135 (97.7)	
Per capita household income in RMB				<0.001
*< 5000*	1110 (44.3)	951 (70.9)	159 (13.7)	
*≥ 5000*	1394 (55.7)	391 (29.1)	1003 (86.3)	
BMI in kg/m^2^				<0.001
*< 18.5*	20 (0.8)	17 (1.3)	3 (0.3)	
*18.5–24*	631 (25.2)	392 (29.2)	239 (20.6)	
*24–28*	1162 (46.4)	596 (44.4)	566 (48.7)	
*≥ 28*	691 (27.6)	337 (25.1)	354 (30.4)	
Smoking history				0.007
*Never*	1696 (67.7)	877 (65.4)	819 (70.5)	
*Ever (current and former)*	808 (32.3)	465 (34.6)	343 (29.5)	
Current drinking status				0.029
*No*	2017 (80.6)	1059 (78.9)	958 (82.4)	
*Yes*	487 (19.4)	283 (21.1)	204 (17.6)	
Scar count				<0.001
*0*	229 (9.1)	190 (14.2)	39 (3.4)	
*1*	1484 (59.3)	808 (60.2)	676 (58.2)	
*≥ 2*	791 (31.6)	344 (25.6)	447 (38.5)	
Self-reported history of immune disorders				0.002
*Yes*	16 (0.6)	2 (0.1)	14 (1.2)	
*No*	2488 (99.4)	1340 (99.9)	1148 (98.8)	
Self-reported history of close contact with tuberculosis patients				0.137
*Yes*	39 (1.6)	26 (1.9)	13 (1.1)	
*No*	2465 (98.4)	1316 (98.1)	1149 (98.9)	
Daily indoor time in hours				<0.001
*< 12*	800 (31.9%)	693 (51.6%)	107 (9.2%)	
*≥ 12*	1704 (68.1%)	649 (48.4%)	1055 (90.8%)	
Daily ventilation time in hours				0.094
*< 0.5*	47 (1.9)	19 (1.4)	28 (2.4)	
*0.5–4*	1451 (57.9)	767 (57.2)	684 (58.9)	
*> 4*	1006 (40.2)	556 (41.4)	450 (38.7)	
Place green plants indoors				<0.001
*Yes*	1314 (52.5)	340 (25.3)	974 (83.8)	
*No*	1190 (47.5)	1002 (74.7)	188 (16.2)	
Using fuel for cooking*				<0.001
*Clean energy*	2298 (91.8)	1157 (86.2)	1141 (98.2)	
*Non-renewable energy sources*	206 (8.2)	185 (13.8)	21 (1.8)	
Using fuel for heating†				<0.001
*Clean energy*	2293 (91.6)	1151 (85.8)	1142 (98.3)	
*Non-renewable energy sources*	211 (8.4)	191 (14.2)	20 (1.7)	

BMI – body mass index

*The fuels used for cooking include clean energy sources such as natural gas and electricity. Non-renewable energy sources for cooking include briquettes, liquid gas in canisters, and wood.

†The fuels used for heating include clean energy sources such as natural gas, electricity and centralised heating. Non-renewable energy sources for heating include coal, oil, wood, *etc.*

### LTBI rates in different regions

**Figure 1:**
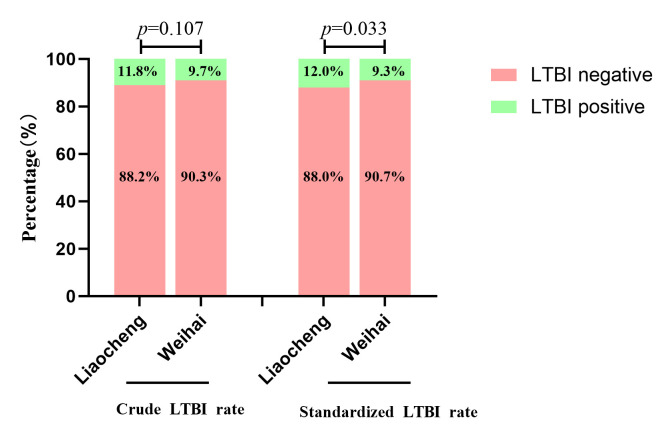
LTBI rate based on the 2020 national population census data. LTBI – latent tuberculosis infection.

Among the entire population, 10.8% (n = 271) of the participants were positive for LTBI (**Table 2**). Among them, the LTBI positive rate in Weihai city was 9.7% (n = 113), and in Liaocheng city was 11.8% (n = 158). There was no statistically significant difference in the LTBI rates between the two regions (*P* = 0.107). After standardising for age and gender using the 2020 national census data, the LTBI positive rate in Weihai city was 9.3%, and in Liaocheng city it was 12.0%, with the difference statistically significant (*P* = 0.033) (**Figure 1**).

**Table 2:** Analysis of the influencing factors of LTBI

Characteristics	n/N (%)	OR (95% CI)†	*P*-value*
Total	271/2504 (10.8)		
Site			0.114
*Liaocheng*	158/1342 (11.8)	1.27 (0.92–1.74)	
*Weihai*	113/1162 (9.7)	Ref.	
Gender			<0.001
*Female*	106/1494 (7.1)	Ref.	
*Male*	165/1110 (14.9)	1.76 (1.14–2.69)	
Age in years			0.822
*< 70*	105/986 (10.6)		
*≥ 70*	166/1518 (10.9)		
Education			0.084
*No schooling*	75/809 (9.3)	Ref.	
*Primary school or higher*	196/1695 (11.6)	1.19 (0.82–1.73)	
Per capita household income in RMB			0.883
*< 5000*	119/1110 (10.7)		
*≥ 5000*	152/1394 (10.9)		
BMI in kg/m^2^			0.967
*< 18.5*	0/20 (0.0)		
*18.5–24*	70/631 (11.1)		
*24–28*	121/1162 (10.4)		
*≥ 28*	80/691 (11.6)		
Smoking history			<0.001
*Never*	143/1696 (8.4)	Ref.	
*Ever (current and former)*	128/808 (15.8)	1.25 (0.84–1.90)	
Current drinking status			<0.001
*No*	197/2017 (9.8)	Ref.	
*Yes*	74/487 (15.2)	1.01 (0.71–1.42)	
Scar count			0.398
*0*	27/229 (11.8)		
*1*	168/1484 (11.3)		
*≥ 2*	76/791 (9.6)		
Self-reported history of immune disorders			0.079
*Yes*	4/16 (25.0)	3.66 (0.99–11.00)	
*No*	267/2488 (10.7)	Ref.	
Self-reported history of close contact with tuberculosis patients			0.004
*Yes*	10/39 (25.6)	3.17 (1.43–6.49)	
*No*	261/2465 (10.6)	Ref.	
Daily indoor time in hours			0.237
*< 12*	78/800 (9.7)		
*≥ 12*	193/1704 (11.3)		
Daily ventilation time in hours			0.004
*< 0.5*	13/47 (27.7)	3.89 (1.57–6.21)	
*0.5–4*	144/1451 (9.9)	Ref.	
*> 4*	114/1006 (11.3)	1.16 (0.89–1.50)	
Place green plants indoors			0.978
*Yes*	142/1314 (10.8)		
*No*	129/1190 (10.8)		
Using fuel for cooking‡			0.441
*Clean energy*	252/2298 (10.9)		
*Non-renewable energy sources*	19/206 (9.2)		
Using fuel for heating§			0.375
*Clean energy*	252/2293 (11.0)		
*Non-renewable energy sources*	19/211 (9.0)		

BMI – body mass index, ref – reference

*Variables that showed statistically significant differences (*P* < 0.2) in the univariate analysis were included in the multivariate analysis model. The *P*-value was the statistical measure used for the χ^2^ test of categorical variables.

†OR (95% CI) represents the results of multivariate analysis, which were adjusted for site, gender, educational level, smoking, drinking, self-reported history of immune diseases, self-reported history of close contact with tuberculosis patients, and daily ventilation time.

‡The fuels used for cooking include clean energy sources such as natural gas and electricity. Non-renewable energy sources for cooking include briquettes, liquid gas in cans, wood, *etc*.

§The fuels used for heating include clean energy sources such as natural gas, electricity and centralised heating. Non-renewable energy sources for heating include coal, oil, wood, *etc*.

### Distribution of air pollutants

**Figure 2:**
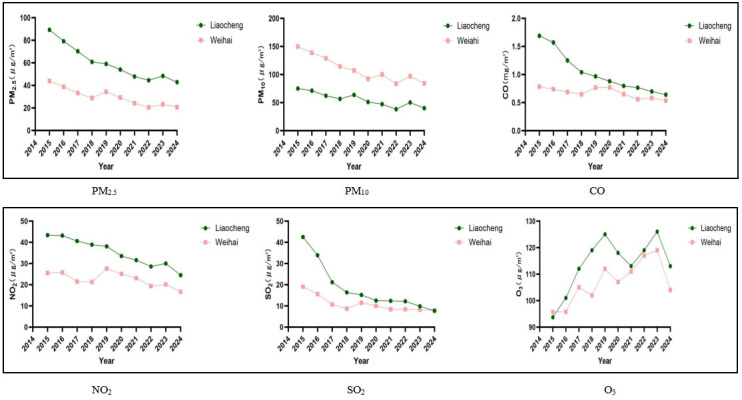
The annual average exposure levels of PM_2.5_, PM_10_, CO, NO_2_, SO_2_, and O_3_ from 2015–2024. CO – carbon monoxide, NO_2_ – nitrogen dioxide, O_3_ – ozone, PM_2.5_ – fine particulate matter, PM_10_ – particulate matter with a diameter of no more than 10 μm, SO_2_ – sulphur dioxide.

Among the six air pollutants, except for O_3_, the concentrations of the remaining five pollutants decreased in Liaocheng and Weihai from 2015–2024. O_3_ exhibited an upward trend annually, yet it also showed a decrease in 2024. The concentrations of the six air pollutants in Liaocheng were generally higher than those in Weihai (**Figure 2**). The reduction rates of SO_2_ and CO in Liaocheng were the highest (Table S1 in the **Online Supplementary Document**). The exposure levels from 2015–2024 were: for PM_2.5_ MD = 58.23 μg/m^3^ (IQR = 29.24–60.10), for PM_10_ MD = 106.35 μg/m^3^ (IQR = 55.26–110.26), for CO MD = 1.02 mg/m^3^ (IQR = 0.68–1.03), for NO_2_ MD = 34.48 μg/m^3^ (IQR = 22.93–35.21), for SO_2_ MD = 18.01μg/m^3^ (IQR = 11.04–18.36) for and O_3_ MD = 113.52 μg/m^3^ (IQR = 107.70–113.85) (Table S1 in the **Online Supplementary Document**).

### The association between air pollutants and the risk of LTBI occurrence

Exposure to air pollutants significantly increased the odds of LTBI. When the exposure period was one and three years, there was a significant positive association between exposure to air pollutants PM_2.5_, CO, and NO_2_, and the odds of LTBI. During the three-year exposure period, for every 10μg/m^3^ increase in PM_2.5_, CO, and NO_2_ concentrations, exposure was associated with 14% (odds ratio (OR) = 1.14; 95% confidence interval (CI) = 1.01–1.30), 3% (OR = 1.03; 95% CI = 1.01–1.05), and 49% higher odds of LTBI detection (OR = 1.49; 95% CI = 1.06–2.08), respectively. As exposure time increases, the correlation between pollutants and LTBI decreases. When the exposure time was extended to 10 years, only CO and NO_2_ remained significantly positively correlated with the odds of LTBI (**Table 3**). In the Liaocheng area, the odds of exposure to PM_2.5_ and NO_2_ were higher than in the Weihai area across various exposure time windows. During the three-year exposure window period, for every 10μg/m^3^ increase in PM_2.5_ and NO_2_ concentrations, the odds in Liaocheng was 29% (OR = 1.35; 95% CI = 1.04–1.78) and 35% (OR = 1.50; 95% CI = 1.05–1.95) higher than that in Weihai (OR = 1.06; 95% CI = 1.03–1.09 and OR = 1.15; 95% CI = 1.02–1.30).

**Table 3:** Association between air pollutants and the risk of LTBI occurrence

Air pollutant	Pollutant exposure in μg/m^3^ (2015–2024)	Model							
	**MD (IQR)**	**1 y, OR (95%CI)**	***P*–value**	**3 y, OR (95%CI)**	***P*–value**	**5 y, OR (95%CI)**	***P*–value**	10 y, OR (95%CI)*	***P*–value**
**Total (n/N = 271/2504)**									
PM_2.5_	58.23 (29.24–60.10)	1.16 (1.01–1.33)	0.038	1.14 (1.01–1.30)	0.041	1.14 (1.00–1.29)	0.046	1.10 (0.99–1.22)	0.057
PM_10_	106.35 (55.26–110.26)	1.07 (1.00–1.15)	0.045	1.07 (1.00–1.15)	0.046	1.07 (0.99–1.14)	0.051	1.06 (0.99–1.12)	0.059
CO, mg/m^3^	1.02 (0.68–1.03)	1.03 (1.00–1.06)	0.026	1.03 (1.01–1.05)	0.011	1.03 (1.00–1.05)	0.018	1.01 (1.00–1.02)	0.045
NO_2_	34.48 (22.93–35.21)	1.50 (1.13–2.18)	0.034	1.49 (1.06–2.08)	0.021	1.48 (1.05–2.10)	0.026	1.30 (1.01–1.66)	0.040
SO_2_	18.01 (11.04–18.36)	0.99 (0.96–1.03)	0.794	1.02 (1.00–1.03)	0.035	1.01 (1.00–1.03)	0.037	1.00 (1.00–1.01)	0.081
O_3_	113.52 (107.70–113.85)	1.45 (1.00–2.11)	0.049	1.30 (0.81–2.08)	0.277	1.41 (0.87–2.28)	0.165	1.54 (0.97–2.44)	0.066
**Liaoceng (n/N = 158/1342)**									
PM_2.5_	60.10 (59.11–60.20)	1.28 (1.00–1.56)	0.046	1.35 (1.04–1.78)	0.027	1.34 (1.02–1.80)	0.044	1.16 (0.93–1.46)	0.213
PM_10_	110.25 (108.51–110.61)	1.14 (0.97–1.36)	0.134	1.16 (0.97–1.39)	0.116	1.12 (0.95–1.35)	0.193	1.03 (0.93–1.14)	0.602
CO, mg/m^3^	1.02 (1.01–1.03)	1.05 (0.84–1.28)	0.442	1.05 (0.99–1.11)	0.701	1.05 (0.99–1.12)	0.715	1.02 (0.97–1.11)	0.586
NO_2_	35.21 (34.93–35.30)	1.56 (1.10–2.02)	0.020	1.50 (1.05–1.95)	0.033	1.27 (1.04–1.51)	0.047	1.13 (0.89–1.37)	0.868
SO_2_	18.35 (18.28–18.46)	1.01 (0.97–1.05)	0.784	0.98 (0.92–1.05)	0.535	1.03 (0.99–1.06)	0.457	1.00 (0.98–1.02)	0.809
O_3_	113.85 (113.71–114.21)	1.57 (1.24–1.90)	0.025	1.38 (1.07–1.70)	0.039	1.04 (0.88–1.20)	0.572	1.09 (0.77–1.42)	0.235
**Weihai (n/N = 113/1162)**									
PM_2.5_	29.24 (28.76–29.28)	1.10 (0.91–1.32)	0.338	1.06 (1.03–1.09)	0.014	1.04 (1.00–1.11)	0.035	0.83 (0.60–1.10)	0.209
PM_10_	55.25 (54.48–55.26)	1.08 (0.92–1.25)	0.368	1.06 (0.90–1.24)	0.447	1.00 (0.81–1.22)	0.837	0.83 (0.64–1.06)	0.646
CO, mg/m^3^	0.67 (0.65–0.68)	1.02 (0.99–1.10)	0.098	1.03 (0.91–1.19)	0.066	1.03 (0.91–1.23)	0.103	1.01 (0.95–1.05)	0.776
NO_2_	22.88 (22.87–23.15)	1.15 (1.00–1.33)	0.046	1.15 (1.02–1.30)	0.029	1.17 (1.02–1.34)	0.021	1.12 (0.98–1.26)	0.067
SO_2_	11.03 (10.33–11.04)	0.98 (0.94–1.03)	0.475	1.03 (0.99–1.06)	0.107	0.99 (0.93–1.06)	0.089	0.99 (0.97–1.02)	0.209
O_3_	107.59 (107.58–107.76)	1.14 (0.95–1.36)	0.163	0.93 (0.82–1.07)	0.306	0.94 (0.80–1.11)	0.468	1.12 (0.93–1.34)	0.237

CI – confidence interval, CO – carbon monoxide, IQR – interquartile range, LTBI – latent tuberculosis infection, MD – median, NO_2_ – nitrogen dioxide, OR – odds ratio, O_3_ – ozone, PM – particulate matter, SO_2_ – sulphur dioxide

*The model adjusted for gender, educational level, smoking status, drinking habits, self-reported history of immune diseases, self-reported history of close contact with tuberculosis patients, and daily ventilation time. The OR (95% CI) represents the risk ratio when the concentrations of PM_2.5_, PM_10_, NO_2_, CO, and O_3_ increase by 10 μg/m^3^, and the concentration of SO_2_ increases by 0.1 μg/m^3^.

The quantile model results indicated that as PM_2.5_ and CO exposure levels increased (Q1–4), there was a significant upward trend in the odds of LTBI (*P* = 0.003 for PM_2.5_ and *P* = 0.030 for CO). The OR values of LTBI corresponding to Q4 compared to Q1 were OR = 1.72 (95% CI = 1.17–2.51) and OR = 1.52 (95% CI = 1.02–2.25), and there was an overall dose-response trend. Overall, a dose-response trend was observed for PM_2.5_ and O_3_ in the Liaocheng area, whereas no such relationship was detected in the Weihai area (Table S1 in the **Online Supplementary Document**).

### The association between comprehensive exposure to air pollutants and the odds of LTBI

The overall results of the weighted pollution model indicated that the WPI was positively correlated with the predicted probability of LTBI infection. Multivariate logistic regression indicates that for every one-unit increase in WPI, the odds of LTBI detection increased by 22% (OR = 1.22; 95% CI = 1.04–1.44, *P* = 0.017). Quantile analysis indicated that the odds in the high-exposure group (Q5) were 1.59 times (95% CI = 1.03–2.49) that of the low-exposure group (Q1), and there was an overall dose-response trend (*P* = 0.025) (Figure S1, Panel A in the **Online Supplementary Document**). For every one-unit increase in WPI, the odds of LTBI detection increased by 68% (OR = 1.68; 95% CI = 1.05–2.78, *P* = 0.035) in Liaocheng and the odds of LTBI detection also showed a 30% increase (OR = 1.30; 95% CI = 0.98–1.76, *P* = 0.077) in the Weihai region, although it did not reach statistical significance. Quantile analysis revealed an overall dose-response trend in Liaocheng (*P* = 0.035) (Figure S1, Panel B in the **Online Supplementary Document**), whereas no such trend was observed in Weihai (*P* = 0.075) (Figure S1, Panel C in the **Online Supplementary Document**).

### Subgroup analysis

We conducted a subgroup analysis using a three-year exposure window. Additionally, based on the contribution levels of each pollutant in the weighted pollution model, we selected four pollutants for the analysis – PM_2.5_, PM_10_, CO, and NO_2_. As household income rose, the intensity of the odds association diminished. In subgroup analyses, we observed a significant interaction between household per capita income and air pollutant exposure, with higher ORs in the lower-income group (< 5000 RMB) than in the higher-income group (≥ 5000 RMB). Specifically, for PM_2.5_ (OR = 1.56; 95% CI = 1.13–2.30 *vs.* OR = 1.13; 95% CI = 0.9–1.36), PM_10_ (OR = 1.26; 95% CI = 1.06–1.54 *vs.* OR = 1.06; 95% CI = 0.96–1.17), CO (OR = 1.08; 95% CI = 1.02–1.16 *vs.* OR = 1.01; 95% CI = 0.99–1.05), and NO_2_ (OR = 3.32; 95% CI = 1.44–5.23 *vs.* OR = 1.45; 95% CI = 0.91–2.31). The interaction with household per capita income was statistically significant (*P* < 0.05). In the subgroup with daily ventilation time < 0.5 hours, the odds of LTBI were higher than those in the subgroups with ventilation times of 0.5–4 hours and > 4 hours. The subgroup using non-renewable energy for cooking had higher odds than the subgroup using clean energy, and the interaction between the four pollutants and this subgroup was statistically significant (*P* < 0.05) (Table S2 in the **Online Supplementary Document**).

## DISCUSSION

To our knowledge, this study is the first to examine the association between multiple air pollutants and the prevalence of LTBI among the elderly population in China. The study found that long-term exposure to PM_2.5_, PM_10_, CO, NO_2_, SO_2_, and O_3_ increases the odds of LTBI, with variations observed among different polluted areas.

In recent years, a growing body of evidence has indicated that air pollutants are closely associated with the incidence and mortality risk of active TB [19]. However, research on their impact on LTBI prevalence remains scarce, despite preliminary evidence from existing studies. A multi-centre cohort study [20] conducted in 2013 among rural populations in China indicated that one-year exposure to PM_2.5_ among rural elderly was associated with an elevated risk of LTBI, although this association was not statistically significant. A study conducted in six prefecture-level cities in Eastern China [21], which included 198 275 students, found that for every 1μg/m^3^ increase in PM_2.5_ and PM10, the odds of LTBI detection increased by 0.82% (95% CI = 0.65–1.00) and 0.91% (95% CI = 0.63–1.20), respectively, one year later. A cross-sectional study of 5193 freshmen from nine universities in Eastern China [22] found that an increase in outdoor PM_10_ concentration was significantly and positively associated with the odds of LTBI (OR = 1.35; 95% CI = 1.10–1.65). This aligns with our research findings and may be associated with various biological mechanisms. First, PM_2.5_ and PM_10_, acting as aerosol carriers, can significantly alter the dynamic characteristics of droplets containing MTB. The latest research from the Singapore Agency for Science, Technology, and Research [23] indicates that in polluted environments, pollutants such as PM_2.5_ and NO_2_ can increase air viscosity, thereby extending the airborne contagious period of MTB-containing droplets by two to three times [24]. This significantly increases the risk window for human exposure to MTB. Second, studies have shown that air pollutants create a physically protective acidic microenvironment for MTB (potential of hydrogen of 4.5–5.2) [25]. This acidic condition actually helps MTB resist environmental pressures and prolongs its survival time. Third, air pollutants continuously induce oxidative stress and inflammatory responses, increasing the production of reactive oxygen species. As oxidants, NO_2_ and O_3_ cause oxidative damage and induce the release of inflammatory cytokines, which further exacerbate the inflammatory response [26].

In the regional stratified analysis, during a three-year exposure window period, this association showed significant regional heterogeneity. This pertains to the varying levels of exposure in different regions. Liaocheng, an industrial city with more severe pollution, was situated in an economically less-developed area [27]. The pollutant concentration was significantly higher than that of Weihai. There was a significant interaction effect between lower household income and outdoor air pollution, whereas the higher economic level of Weihai might serve as a protective factor, mitigating the negative impact of air pollution [28]. At the same time, pollutant distribution in Liaocheng was widespread, but concentrations were low and had a limited range, making it challenging to detect significant differences [29]. Furthermore, the OR value for the correlation between air pollution and LTBI was close to 1.0. Given that air pollutants were universal exposure factors affecting the entire population, this small effect association still holds significant public health value. For large-scale populations, even a slight increase in the risk of disease onset could lead to a significant increase in disease burden. When conducting a stratified analysis, a reduction in sample size can diminish statistical power. Even if there was a comparable level of actual association in Weihai, the current stratified sample size might not be adequate to establish statistical significance. In conclusion, the significant associations of PM_2.5_, PM_10_, CO, NO_2_, and SO_2_ observed in the overall analysis were no longer evident after stratified analysis, suggesting that the impact of air pollution on the odds of LTBI is strongly influenced by regional factors. The health benefits of controlling air pollution may be most significant in areas with high levels of pollution.

Meanwhile, the study found that the association between air pollutants and LTBI weakened as the exposure time increased across different exposure windows. This is likely because LTBI is more strongly affected by short-term acute exposure to air pollutants. Short-term exposure (*e.g.* one year) can directly damage the respiratory tract mucosal barrier, inhibit the function of alveolar macrophages, and trigger the release of pro-inflammatory factors (*e.g.* interleukin-6 and tumour necrosis factor-alpha), weaken the T-cell immune response, thereby promoting the activation of LTBI or the conversion to IGRA positivity [30]. With long-term exposure, the body may develop immune tolerance, and most people adapt to a certain concentration of air pollutants, leading to reduced sensitivity and exhibiting a non-significant correlation phenomenon [29]. A study conducted in Shandong Province [31] revealed that O_3_ exhibited the strongest correlation with drug-resistant TB within a 90-day exposure period. However, the strength of this association diminished over longer exposure windows due to fluctuations in concentration.

In this study, we recommend adding air pollutant concentrations to TB prevention and control risk assessments. For areas with pollutant levels (*e.g.* PM_2.5_, PM_10_) exceeding national standards, enforce air quality controls and expand LTBI screening, prioritising regular checks for vulnerable groups such as the elderly, to achieve dual prevention through environmental management and population screening. Moreover, establish an integrated environmental and TB monitoring system to develop predictive risk maps, and integrate real-time data from environmental and disease control departments to inform evidence-based TB prevention and control strategies.

### Limitations

This study has several limitations. First, potential exposure misclassification bias exists in the exposure assessment: this study used ambient air pollutant concentrations at residential addresses as surrogate exposure indicators and did not quantitatively analyse indoor air pollution exposure. This may lead to biased effect estimates for the association between exposure and outcome toward the null value. Future studies could employ personal wearable monitoring devices to conduct more accurate individual exposure assessments, thereby reducing exposure misclassification bias. Second, this study adopted a cross-sectional design, which only allowed for the identification of a statistical association between ambient air pollutant exposure and LTBI detection. It could not establish a causal relationship or confirm the temporal sequence between exposure and MTB infection. Prospective cohort studies with long-term follow-up are warranted to elucidate the potential causal link between air pollutant exposure and LTBI risk. Third, despite adjusting for multiple potential confounders in the multivariate model, the influence of unknown, unmeasured confounders cannot be entirely ruled out. Separating socioeconomic factors from the effects of air pollution exposure is challenging. The survey subjects may include family members with similar living environments and mutually influencing behaviours, which could lead to intrinsic confounding. Fourth, this study adopted single-pollutant models to circumvent multicollinearity among air pollutants; however, potential spatial autocorrelation was not explicitly addressed in the statistical analyses. Future studies could incorporate spatial statistical models and integrate geographic variables to adjust for spatial confounding, thereby enabling a more precise analysis of the association between ambient air pollution and LTBI. Finally, the effect sizes observed in this study were relatively modest, and the corresponding confidence intervals were wide. This pattern is likely attributable to residual confounding factors and the relatively small sample size, which together led to insufficient statistical power. Future studies with larger, more representative samples are warranted to validate these findings and confirm the observed associations.

The generalisability of this study's results to other populations requires careful consideration. First, the study focused exclusively on the elderly, whose immunological vulnerability and unique lifestyle patterns may have strengthened the identified association between air pollution and LTBI. Consequently, the conclusions might not be directly applicable to young and middle-aged groups or to elderly populations in regions with vastly different lifestyles and levels of protection. Second, while the study areas were characterised by combined PM_2.5_ and PM_10_ pollution, pollutant sources and compositions differ markedly across high-pollution regions. The effects of industrial exhaust, construction dust, and other forms of pollution on LTBI remain unclear, necessitating cautious interpretation of the conclusions.

## CONCLUSIONS

We revealed a significant correlation between air pollutants and the prevalence of LTBI. The research found that long-term exposure to air pollutants significantly increases the odds of LTBI in Chinese cities, and shows regional differences. This underscores the importance of controlling air pollution in TB prevention and control and provides a scientific basis for formulating relevant public health policies.

## Additional material


Online Supplementary Document
