# Supplementary material for: The association between exposure to air pollutants and latent tuberculosis infection prevalence in the elderly population: a population-based cross-sectional study from China
Source: J Glob Health. 2026 Apr 3;16:04116. doi: 10.7189/jogh.16.04116 (PMC13047348; doi:10.7189/jogh.16.04116)
Supplement: Online Supplementary Document [file jogh-16-04116-s001.pdf]

Supplement to: Cite as: Zhao Y, Li H, He Y, Guo X, Jin J, Li T, Wang S, Cao X, Deng Y, Feng B, Li Z, Wang Q, Cao S, Zhao Z, Zhang X, Zhu A, Liang J, Geng H, Sang Y, Li J, Wang L, Fan X, Xin H, Du J, Cao X, Zhang Z, Gao L. The association between exposure to air pollutants and latent tuberculosis infection prevalence in the elderly population: a population-based cross-sectional study from China. J Glob Health. 2026;16:04116.

Table S1. Pollutant quartiles and the risk of latent tuberculosis infection*

Air pollutant	Quartiles 1	Quartiles 2	Quartiles 3	Quartiles 4	*p* for trend†
**Total (271/2504)**					
**$PM_{2.5}$**	Reference	0.78 (0.53-1.14)	1.03 (0.70-1.53)	1.72 (1.17-2.51)	**0.003**
**PM_{10}**	Reference	0.85 (0.58-1.26)	1.31 (0.91-1.18)	1.09 (0.72-1.63)	0.310
**CO**	Reference	1.02 (0.70-1.49)	1.22 (0.83-1.80)	1.52 (1.02-2.25)	**0.030**
**NO_2**	Reference	0.78 (0.52-1.16)	1.15 (0.80-1.66)	1.27 (0.86-1.87)	0.086
**SO_2**	Reference	0.88 (0.61-1.27)	1.45 (0.99-2.11)	1.03 (0.79-1.27)	0.826
**O_3**	Reference	0.74 (0.46-1.14)	1.45 (1.02-2.05)	0.95 (0.64-1.39)	0.452
**Liaocheng (158/1342)**					
**$PM_{2.5}$**	Reference	1.14 (0.77-1.89)	0.95 (0.63-1.59)	2.03 (1.29-3.22)	**0.009**
**PM_{10}**	Reference	1.60 (1.04-2.50)	1.34 (0.87-2.29)	1.29 (0.77-1.81)	0.801
**CO**	Reference	0.93 (0.50-1.36)	0.86 (0.44-1.28)	1.11 (0.71-1.73)	0.797
**NO_2**	Reference	0.81 (0.51-1.30)	1.09 (0.70-1.56)	1.05 (0.82-1.28)	0.257
**SO_2**	Reference	1.07 (0.70-1.65)	0.97 (0.74-1.21)	0.95 (0.84-1.06)	0.057
**O_3**	Reference	0.99 (0.70-1.55)	1.29 (1.08-2.50)	1.61 (1.23-1.99)	**0.030**
**Weihai (113/1162)**					
**$PM_{2.5}$**	Reference	1.51 (0.79-2.76)	1.09 (0.69-1.72)	1.06 (0.67-1.45)	0.063
**PM_{10}**	Reference	1.56 (0.95-2.64)	1.23 (0.66-2.30)	0.89 (0.43-1.79)	0.609
**CO**	Reference	0.99 (0.52-1.81)	1.23 (0.78-1.91)	1.19 (0.85-1.53)	0.263
**NO_2**	Reference	1.89 (1.15-3.20)	1.33 (0.72-2.46)	1.35 (0.99-1.71)	0.358
**SO_2**	Reference	1.15 (0.99-1.31)	1.05 (0.68-1.61)	0.83 (0.43-1.49)	0.546
**O_3**	Reference	1.29 (0.83-2.03)	1.13 (0.78-1.48)	0.89 (0.67-1.11)	0.240

CO: Carbon monoxide; $PM_{2.5}$: Fine particulate matter; NO_2: Nitrogen dioxide; O_3: Ozone; PM_{10}: Particulate matter with a diameter of no more than 10 μm; SO_2: Sulfur dioxide.

* The quartiles of pollutants were classified based on the pollutant exposure levels from 2022 to 2024. The model adjusted for gender, education level, smoking, drinking, self-reported history of immune diseases, self-reported history of close contact with tuberculosis patients, and daily ventilation time.

† The *p*-value represents the result of the trend test.

STROBE Statement—Checklist of items that should be included in reports of ***cross-sectional studies***

	Item No	Recommendation	Page No
**Title and abstract**	1	(*a*) Indicate the study's design with a commonly used term in the title or the abstract	1-2
		(*b*) Provide in the abstract an informative and balanced summary of what was done and what was found	2-3
**Introduction**			
Background/rationale	2	Explain the scientific background and rationale for the investigation being reported	3-4
Objectives	3	State specific objectives, including any prespecified hypotheses	3-4
**Methods**			
Study design	4	Present key elements of study design early in the paper	4-5
Setting	5	Describe the setting, locations, and relevant dates, including periods of recruitment, exposure, follow-up, and data collection	4-5
Participants	6	(*a*) Give the eligibility criteria, and the sources and methods of selection of participants	4-5
Variables	7	Clearly define all outcomes, exposures, predictors, potential confounders, and effect modifiers. Give diagnostic criteria, if applicable	5-6
Data sources/ measurement	8*	For each variable of interest, give sources of data and details of methods of assessment (measurement). Describe comparability of assessment methods if there is more than one group	6-7
Bias	9	Describe any efforts to address potential sources of bias	6-7
Study size	10	Explain how the study size was arrived at	7-8
Quantitative variables	11	Explain how quantitative variables were handled in the analyses. If applicable, describe which groupings were chosen and why	7-8
Statistical methods	12	(*a*) Describe all statistical methods, including those used to control for confounding	7-8
		(*b*) Describe any methods used to examine subgroups and interactions	7-8
		(*c*) Explain how missing data were addressed	7-8
		(*d*) If applicable, describe analytical methods taking account of sampling strategy	NA
		(*e*) Describe any sensitivity analyses	7-8
**Results**			
Participants	13*	(a) Report numbers of individuals at each stage of study—eg numbers potentially eligible, examined for eligibility, confirmed eligible, included in the study, completing follow-up, and analysed	9
		(b) Give reasons for non-participation at each stage	9
		(c) Consider use of a flow diagram	NA
Descriptive data	14*	(a) Give characteristics of study participants (eg demographic, clinical, social) and information on exposures and potential confounders	9-10
		(b) Indicate number of participants with missing data for each variable of interest	9-10
Outcome data	15*	Report numbers of outcome events or summary measures	9-10
Main results	16	(*a*) Give unadjusted estimates and, if applicable, confounder-adjusted estimates and their precision (eg, 95% confidence interval). Make clear which confounders were adjusted for and why they were included	9-11

		(*b*) Report category boundaries when continuous variables were categorized	9-11
		(*c*) If relevant, consider translating estimates of relative risk into absolute risk for a meaningful time period	NA
Other analyses	17	Report other analyses done—eg analyses of subgroups and interactions, and sensitivity analyses	11
**Discussion**			
Key results	18	Summarise key results with reference to study objectives	12
Limitations	19	Discuss limitations of the study, taking into account sources of potential bias or imprecision. Discuss both direction and magnitude of any potential bias	15
Interpretation	20	Give a cautious overall interpretation of results considering objectives, limitations, multiplicity of analyses, results from similar studies, and other relevant evidence	12-15
Generalisability	21	Discuss the generalisability (external validity) of the study results	16
**Other information**			
Funding	22	Give the source of funding and the role of the funders for the present study and, if applicable, for the original study on which the present article is based	17

*Give information separately for exposed and unexposed groups.

Note: An Explanation and Elaboration article discusses each checklist item and gives methodological background and published examples of transparent reporting. The STROBE checklist is best used in conjunction with this article (freely available on the Web sites of PLoS Medicine at http://www.plosmedicine.org/, Annals of Internal Medicine at http://www.annals.org/, and Epidemiology at http://www.epidem.com/). Information on the STROBE Initiative is available at www.strobe-statement.org.

A

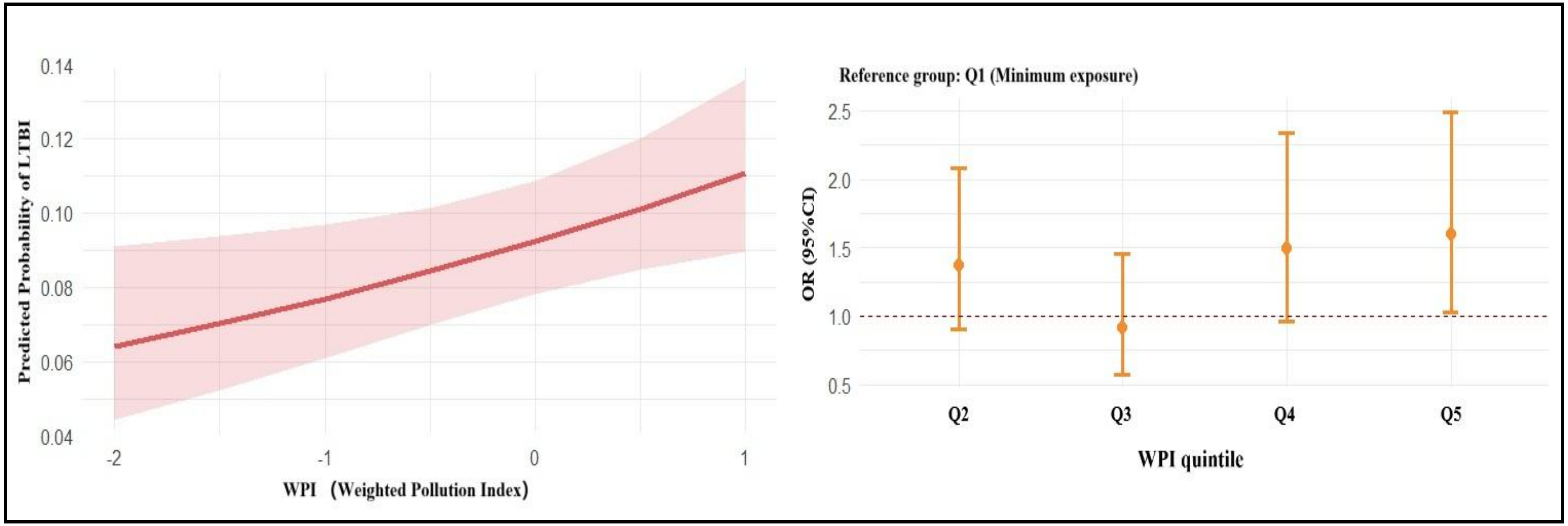

0.14
0.12
0.10
0.08
0.06
0.04
Predicted Probability of LTBI
-2
-1
0
1
WPI （Weighted Pollution Index）
Reference group: Q1 (Minimum exposure)
2.5
2.0
1.5
1.0
0.5
OR (95%CI)
Q2
Q3
Q4
Q5
WPI quintile

B

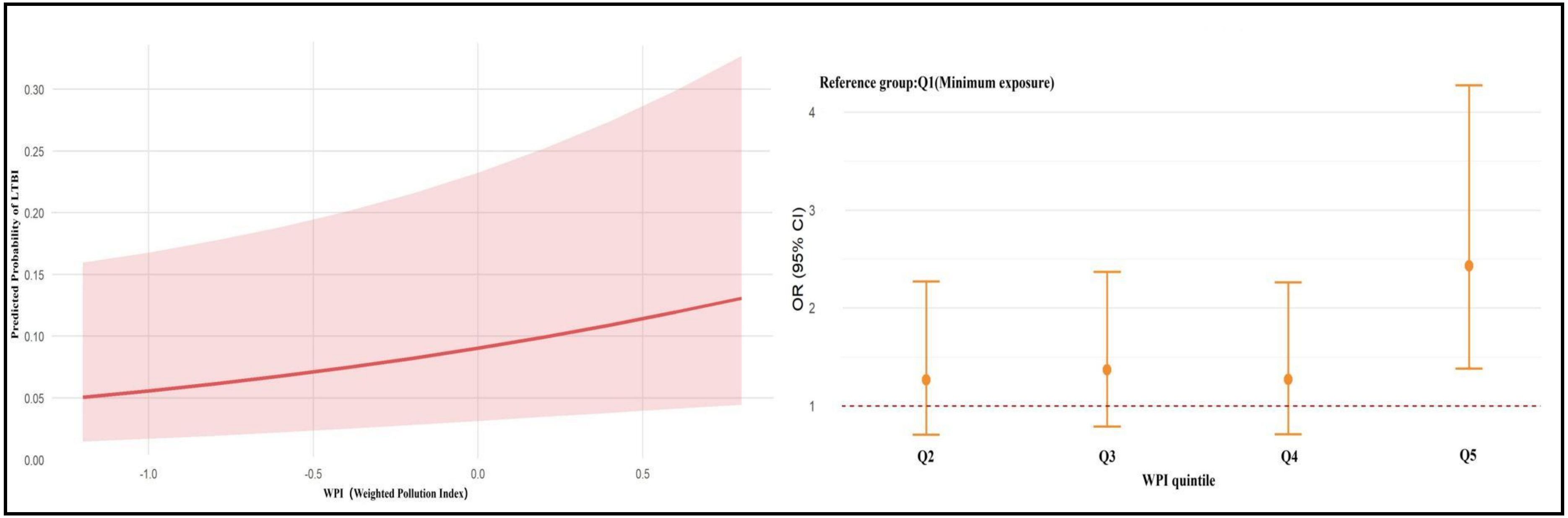
Predicted Probability of LTBI
0.00
0.05
0.10
0.15
0.20
0.25
0.30
-1.0
-0.5
0.0
0.5
WPI（Weighted Pollution Index）
Reference group:Q1(Minimum exposure)
OR (95% CI)
1
2
3
4
Q2
Q3
Q4
Q5
WPI quintile

C

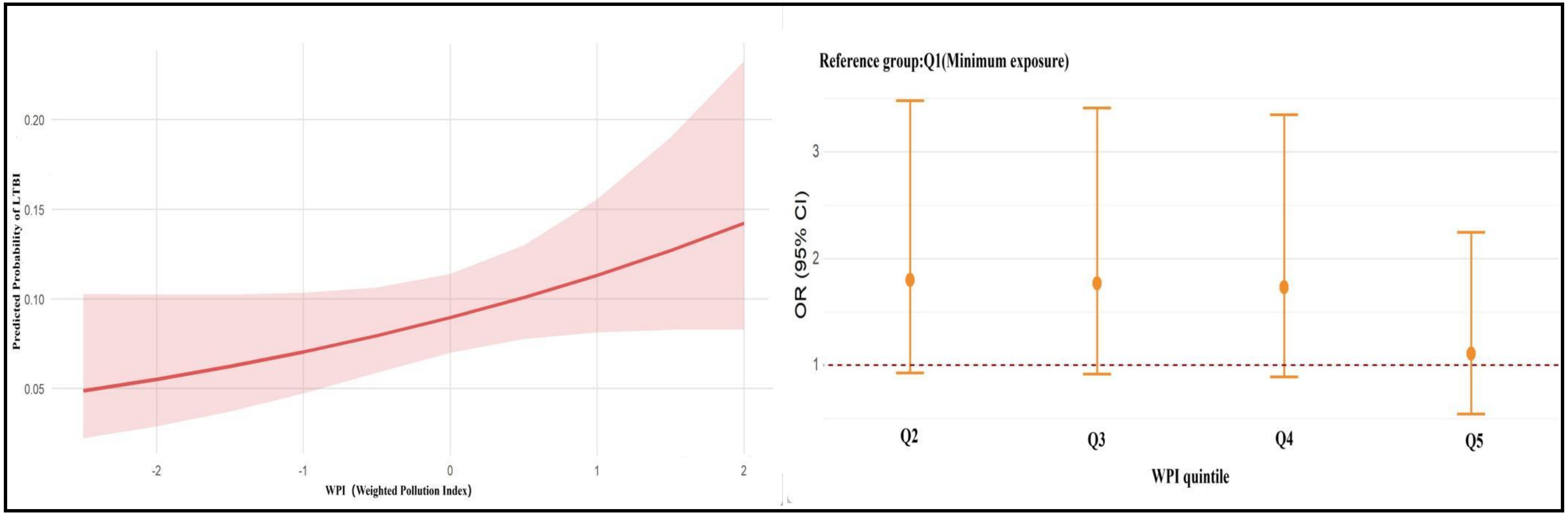

Figure S1. Prediction probability and quantile model of LTBI based on weighted pollution model [a]

WPI:weighed pollution index,standardize , $PM_{2.5}$, PM_{10}, CO, NO_2, SO_2 and O_3 using Z-score. For the overall model A, the trend test of the quantile model shows p =0.025. For the model of Liaocheng area B, the trend test of the quantile model also shows p =0.035 For the model of Weihai area C, the trend test of the quantile model shows p = 0.075.

* The weighted pollution model refers to the pollutant exposure levels during the period from 2022 to 2024. The model adjusted for factors such as gender, education level, smoking, drinking, self-reported history of immune diseases, self-reported history of close contact with tuberculosis patients, and daily ventilation time.

Table S2. Subgroup analysis of the association between air pollution exposure and the risk of LTBI occurrence*

Characteristics	$PM_{2.5}$		PM_{10}		CO		NO_2	
	OR (95%CI)	†$p_{interaction}$	OR (95%CI)	$p_{interaction}$	OR (95%CI)	$p_{interaction}$	OR (95%CI)	$p_{interaction}$
**Total**	1.14 (1.01-1.30)		1.07 (1.00-1.15)		1.03 (1.01-1.05)		1.49 (1.06-2.08)	
**Gender**		0.359		0.396		0.641		0.682
Female	1.32 (1.02-1.69)		1.15 (1.01-1.31)		1.04 (1.00-1.08)		1.82 (0.95-3.43)	
Male	1.09 (0.94-1.27)		1.05 (0.97-1.13)		1.02 (0.99-1.05)		1.40 (0.95-2.09)	
**Age**		0.967		0.982		0.821		0.833
<70 years	1.12 (0.91-1.37)		1.06 (0.95-1.18)		1.03 (0.99-1.06)		1.52 (0.89-2.59)	
⩾70 years	1.14 (0.97-1.36)		1.07 (0.98-1.17)		1.03 (1.00-1.05)		1.44 (0.94-2.23)	
**Education**		0.694		0.843		0.776		0.689
No schooling	1.49 (0.80-3.76)		1.18 (0.87-1.88)		1.02 (0.95-1.13)		1.35 (0.41-6.29)	
Primary school or higher	1.14 (0.99-1.30)		1.07 (0.99-1.15)		1.03 (1.01-1.05)		1.53 (1.08-2.16)	
**Per capita household income**		0.033		0.039		0.032		0.037
<5000 RMB	1.56 (1.13-2.30)		1.26 (1.06-1.54)		1.08 (1.02-1.16)		3.32 (1.44-8.89)	
⩾5000 RMB	1.13 (0.93-1.36)		1.06 (0.96-1.17)		1.01 (0.99-1.05)		1.45 (0.91-2.31)	
**BMI**		0.752		0.749		0.558		0.553
<18.5 kg/m^2	NA		NA		NA		NA	
18.5 - <24 kg/m^2	1.19 (0.91-1.56)		1.09 (0.95-1.26)		1.04 (0.99-1.09)		1.86 (0.93-3.85)	

24 - <28 kg/m^2	1.13 (0.93-1.36)		1.06 (0.96-1.17)		1.02 (0.99-1.05)		1.32 (0.80-2.17)	
⩾28 kg/m^2	1.20 (0.94-1.54)		1.11 (0.97-1.26)		1.03 (0.99-1.07)		1.80 (0.96-3.38)	
**Smoking history**		0.260		0.280		0.559		0.541
Never	1.05 (0.88-1.24)		1.02 (0.94-1.12)		1.02 (0.99-1.05)		1.29 (0.82-2.04)	
Ever(current and former)	1.29 (1.06-1.56)		1.14 (1.03-1.26)		1.04 (1.01-1.07)		1.82 (1.11-2.99)	
**Current drinking status**		0.232		0.253		0.519		0.430
No	1.05 (0.84-1.31)		1.03 (0.91-1.16)		1.02 (0.98-1.06)		1.32 (0.74-2.40)	
Yes	1.19 (1.01-1.39)		1.09 (1.00-1.19)		1.03 (1.00-1.05)		1.57 (1.04-2.36)	
**Scar count**		0.001		0.003		0.131		0.192
0	1.64 (1.41-1.89)		NA		1.18 (1.04-1.45)		NA	
1	1.09 (0.93-1.29)		1.05 (0.96-1.14)		1.02 (0.99-1.05)		1.39 (0.91-2.13)	
≥2	1.06 (0.83-1.34)		1.03 (0.91-1.17)		1.02 (0.98-1.06)		1.29 (0.69-2.38)	
**Self-reported history of immune disorders**		0.233		0.203		0.908		0.649
Yes	NA		NA		1.54 (0.90-4.49)		NA	
No	1.15 (1.01-1.31)		1.07 (1.00-1.15)		1.03 (1.01-1.05)		1.50 (1.07-2.10)	
**Self-reported history of close contact with tuberculosis patients**		0.085		0.081		0.074		0.094
Yes	3.21 (1.23-12.30)		1.84 (1.12-3.72)		1.26 (1.04-1.66)		NA	
No	1.13 (0.99-1.28)		1.06 (0.99-1.14)		1.02 (1.00-1.05)		1.45 (1.03-2.03)	

**Daily indoor time**		0.317		0.322		0.275		0.514
<12 hours	1.41 (0.99-2.13)		1.20 (0.99-1.49)		1.07 (1.00-1.15)		2.17 (0.92-4.64)	
≥12 hours	1.21 (1.03-1.42)		1.10 (1.01-1.20)		1.04 (1.01-1.06)		1.82 (1.19-2.77)	
**Daily ventilation time**		0.007		0.008		0.004		0.006
< 0.5 hours	2.24 (1.12-4.84)		1.53 (1.06-2.30)		1.16 (1.03-1.35)		NA	
0.5-4 hours	1.05 (0.87-1.25)		1.02 (0.93-1.12)		1.01 (0.98-1.04)		1.15 (0.73-1.83)	
> 4 hours	1.21 (0.99-1.48)		1.11 (0.99-1.23)		1.04 (1.01-1.07)		1.81 (1.08-3.06)	
**Place green plants indoors**		0.568		0.534		0.552		0.424
Yes	1.31 (1.09-1.58)		1.15 (1.04-1.27)		1.05 (1.02-1.08)		2.25 (1.38-3.65)	
No	1.10 (0.87-1.42)		1.05 (0.92-1.20)		1.02 (0.98-1.06)		1.30 (0.71-2.49)	
**Using fuel for cooking‡**		0.048		0.048		0.028		0.029
Non-renewable energy sources	1.17 (1.02-1.33)		1.08 (1.01-1.16)		1.03 (1.01-1.05)		1.59 (1.12-2.25)	
Clean energy	0.90 (0.50-1.77)		0.94 (0.69-1.36)		0.98 (0.91-1.06)		0.66 (0.17-3.03)	
**Using fuel for heating§**		0.263		0.251		0.415		0.375
Non-renewable energy sources	1.17 (1.03-1.34)		1.09 (1.01-1.16)		1.03 (1.01-1.05)		1.57 (1.12-2.23)	
Clean energy	0.93 (0.47-2.09)		0.95 (0.66-1.45)		1.00 (0.91-1.14)		0.89 (0.18-6.51)	

BMI：body mass index; CO: Carbon monoxide; $PM_{2.5}$: Fine particulate matter; LTBI: Latent Tuberculosis Infection; NO_2: Nitrogen dioxide; NA：Not Available；O_3: Ozone; PM_{10}: Particulate matter with a diameter of no more than 10 μm; RMB：renminbi；SO_2: Sulfur dioxide.The absence of results in the subgroup analysis was due to insufficient patient numbers, which prevented the model from converging.

* The model adjusted for gender, educational level, smoking, drinking, self-reported history of immune diseases, self-reported history of close contact with tuberculosis

patients, and daily ventilation time. The OR (95% CI) represents the risk ratio when the exposure levels of $PM_{2.5}$, PM_{10}, NO_2, and CO pollutants increase by 10 μg/m³ from 2022 to 2024.

† The p-interaction represents the *p*-value of the likelihood ratio test.

‡ The fuels used for cooking include clean energy sources such as natural gas and electricity. Non-renewable energy sources for cooking include briquettes, liquid gas in cans, wood, etc.

§ The fuels used for heating include clean energy sources such as natural gas, electricity and centralized heating. Non-renewable energy sources for heating include coal, oil, wood, etc.

The *p*-value description of the interaction between the covariate and the pollutants (Table S2)：

Per capita household income: $PM_{2.5}$ $p_{interaction}$=0.033、PM_{10} $p_{interaction}$=0.039、CO $p_{interaction}$=0.032、NO_2 $p_{interaction}$=0.037；

Daily ventilation time：$PM_{2.5}$ $p_{interaction}$=0.007、PM_{10} $p_{interaction}$=0.008、CO $p_{interaction}$=0.004、NO_2 $p_{interaction}$=0.006；

Using fuel for cooking：$PM_{2.5}$ $p_{interaction}$=0.048、PM_{10} $p_{interaction}$=0.048、CO $p_{interaction}$=0.028、NO_2 $p_{interaction}$=0.029.

STROBE Statement—Checklist of items that should be included in reports of ***cross-sectional studies***

	Item No	Recommendation	Page No
**Title and abstract**	1	(*a*) Indicate the study's design with a commonly used term in the title or the abstract	1-2
		(*b*) Provide in the abstract an informative and balanced summary of what was done and what was found	2-3
**Introduction**			
Background/rationale	2	Explain the scientific background and rationale for the investigation being reported	3-4
Objectives	3	State specific objectives, including any prespecified hypotheses	3-4
**Methods**			
Study design	4	Present key elements of study design early in the paper	4-5
Setting	5	Describe the setting, locations, and relevant dates, including periods of recruitment, exposure, follow-up, and data collection	4-5
Participants	6	(*a*) Give the eligibility criteria, and the sources and methods of selection of participants	4-5
Variables	7	Clearly define all outcomes, exposures, predictors, potential confounders, and effect modifiers. Give diagnostic criteria, if applicable	5-6
Data sources/ measurement	8*	For each variable of interest, give sources of data and details of methods of assessment (measurement). Describe comparability of assessment methods if there is more than one group	6-7
Bias	9	Describe any efforts to address potential sources of bias	6-7
Study size	10	Explain how the study size was arrived at	7-8
Quantitative variables	11	Explain how quantitative variables were handled in the analyses. If applicable, describe which groupings were chosen and why	7-8
Statistical methods	12	(*a*) Describe all statistical methods, including those used to control for confounding	7-8
		(*b*) Describe any methods used to examine subgroups and interactions	7-8
		(*c*) Explain how missing data were addressed	7-8
		(*d*) If applicable, describe analytical methods taking account of sampling strategy	NA
		(*e*) Describe any sensitivity analyses	7-8
**Results**			
Participants	13*	(a) Report numbers of individuals at each stage of study—eg numbers potentially eligible, examined for eligibility, confirmed eligible, included in the study, completing follow-up, and analysed	9
		(b) Give reasons for non-participation at each stage	9
		(c) Consider use of a flow diagram	NA
Descriptive data	14*	(a) Give characteristics of study participants (eg demographic, clinical, social) and information on exposures and potential confounders	9-10
		(b) Indicate number of participants with missing data for each variable of interest	9-10
Outcome data	15*	Report numbers of outcome events or summary measures	9-10
Main results	16	(*a*) Give unadjusted estimates and, if applicable, confounder-adjusted estimates and their precision (eg, 95% confidence interval). Make clear which confounders were adjusted for and why they were included	9-11

		(*b*) Report category boundaries when continuous variables were categorized	9-11
		(*c*) If relevant, consider translating estimates of relative risk into absolute risk for a meaningful time period	NA
Other analyses	17	Report other analyses done—eg analyses of subgroups and interactions, and sensitivity analyses	11
**Discussion**			
Key results	18	Summarise key results with reference to study objectives	12
Limitations	19	Discuss limitations of the study, taking into account sources of potential bias or imprecision. Discuss both direction and magnitude of any potential bias	15
Interpretation	20	Give a cautious overall interpretation of results considering objectives, limitations, multiplicity of analyses, results from similar studies, and other relevant evidence	12-15
Generalisability	21	Discuss the generalisability (external validity) of the study results	16
**Other information**			
Funding	22	Give the source of funding and the role of the funders for the present study and, if applicable, for the original study on which the present article is based	17

*Give information separately for exposed and unexposed groups.

Note: An Explanation and Elaboration article discusses each checklist item and gives methodological background and published examples of transparent reporting. The STROBE checklist is best used in conjunction with this article (freely available on the Web sites of PLoS Medicine at http://www.plosmedicine.org/, Annals of Internal Medicine at http://www.annals.org/, and Epidemiology at http://www.epidem.com/). Information on the STROBE Initiative is available at www.strobe-statement.org.
